# Detection of Changes in Total Antioxidant Capacity, the Content of Polyphenols, Caffeine, and Heavy Metals of Teas in Relation to Their Origin and Fermentation

**DOI:** 10.3390/foods10081821

**Published:** 2021-08-06

**Authors:** Alica Bobková, Alžbeta Demianová, Ľubomír Belej, Ľuboš Harangozo, Marek Bobko, Lukáš Jurčaga, Katarína Poláková, Monika Božiková, Matúš Bilčík, Július Árvay

**Affiliations:** 1 Department of Food Hygiene and Safety, Faculty of Biotechnology and Food Sciences, Slovak University of Agriculture in Nitra, Trieda Andreja Hlinku 2, 94976 Nitra, Slovakia; alica.bobkova@uniag.sk (A.B.); lubomir.belej@uniag.sk (Ľ.B.); xpolakova@uniag.sk (K.P.); 2 Department of Chemistry, Faculty of Biotechnology and Food Sciences, Slovak University of Agriculture in Nitra, Trieda Andreja Hlinku 2, 94976 Nitra, Slovakia; lubos.harangozo@uniag.sk (Ľ.H.); julius.arvay@uniag.sk (J.Á.); 3 Department of Technology and the Quality of Animal Products, Faculty of Biotechnology and Food Sciences, Slovak University of Agriculture in Nitra, Trieda Andreja Hlinku 2, 94976 Nitra, Slovakia; marek.bobko@uniag.sk (M.B.); xjurcaga@uniag.sk (L.J.); 4 Department of Physics, Faculty of Engineering, Slovak University of Agriculture in Nitra, Trieda Andreja Hlinku 2, 94976 Nitra, Slovakia; monika.bozikova@uniag.sk (M.B.); matus.bilcik@uniag.sk (M.B.)

**Keywords:** fermented tea, non-fermented tea, heavy metals, bioactive compounds, geographical origin identification, linear discriminant analysis, spectrophotometry, AAS, HPLC-DAD

## Abstract

Tea (*Camellia sinensis)* is widely sought for beverages worldwide. Heavy metals are often the main aims of the survey of teas, given that the use of agricultural fertilization is very frequent. Some of these may affect the content of bioactive compounds. Therefore, in this study, we analyzed fermented and non-fermented teas of a single plant origin from Japan, Nepal, Korea, and China, and described mutual correlations and changes in the total antioxidant capacity (TAC), and the content of polyphenols (TPC), caffeine, and heavy metals in tea leaves, in relation to the origin and fermentation process. Using UV-VIS spectrophotometry and HPLC-DAD, we determined variations in bioactive compounds’ content in relation to the fermentation process and origin and observed negative correlations between TAC and TPC. Heavy metal content followed this order: Mn > Fe > Cu > Zn > Ni > Cr > Pb > Co > Cd > Hg. Given the homogenous content of these elements in relation to fermentation, this paper also describes the possibility of using heavy metals as determinants of geographical origin. Linear Discriminant Analysis showed an accuracy of 75% for Ni, Co, Cd, Hg, and Pb, explaining 95.19% of the variability between geographical regions.

## 1. Introduction

A tea infusion prepared from *Camellia sinensis* is one of the most popular beverages worldwide [1,2,3]. Based on the different technological processes, we recognize green and yellow teas as non-fermented, oolong and white teas as semifermented, and black teas as fully fermented [4]. During the oxidation process connected with the fermentation of black tea, oxidation products such as theaflavins and thearubigins are produced. These represent the main difference between black and green tea. Research has shown that black tea has approximately 20–30% *Camellia sinensis* polyphenol content [5].

Phenolic compounds, which are ubiquitous in plants, are an essential part of the human diet and are of considerable interest due to their antioxidant properties and potential beneficial health effects [6]. Total antioxidant capacity (TAC) has become a very popular quality parameter of food products and plant extracts because studies are establishing a link between dietary TAC, as a measure of antioxidant intake, and health [7].

Food consumption has been identified as the primary pathway for human exposure to environmental contaminants [8]. Tea consumption can also be a source of increased levels of toxic trace metals [9]. However, the effects of geological differences on the distribution of heavy metals in soils and their accumulation in tea leaves remain unclear and limited [10].

Depending on the origin of tea leaves, heavy metals accumulation can be derived naturally by soil contamination, pesticides and fertilizers. Studies have shown that trace metals can be introduced into tea products during the fermentation and drying processes of production [11,12].

Given the popularity of tea and limited knowledge in the area mentioned above, our research focuses on detecting the possible effect of the fermentation process on the bioactive compounds and the content of heavy metals in tea leaves originated from one plant. Based on the consistent behavior of heavy metals relative to processing, we focused on using these elements as parameters that are suitable for geographical origin identification, as well as using advanced statistical approaches. Given the hypothesis that the content of selected heavy metals may affect the content of bioactive compounds, we created a correlation between these parameters.

## 2. Materials and Methods

### 2.1. Tea Samples and Infusion Preparation

Samples of eight different green and black teas (each pair originated from the same plant) were obtained from Čajovňa dobrých ľudí, Ltd. (Nitra, Slovakia). Tea samples are described in Table 1. Dry tea leaves were homogenized at 10,000 rpm in a grinder to obtain the fine powder. A quantity of 1 g of the powder was weighted directly into glass beakers, 100 mL of distilled water heated at 99 °C was poured into the sample, and the content was appropriately stirred. Samples were extracted for 10 min and then filtered using KA-1 filter paper (Papírna Perštejn, Ltd., Perštejn, Czech Republic), and extracts were used directly for TAC and TPC. An amount of 5 mL was also filtered through the Q-Max RR syringe filters (0.45 μm PVDF, Frisenette ApS, Denmark) and used for HPLC-DAD analysis.

### 2.2. PH Values

Values of pH were determined using pH meter Testo 206—pH2 (Testo, Lenzkirch, Germany); measurements were performed in beverages at a temperature of 25 °C, in 3 replicates for each sample.

### 2.3. Moisture

The moisture content of tea samples was determined using the KERN DAB 100-3 moisture analyzer (KERN & SOHN GmbH, Balingen, Germany) and expressed as a percentage of moisture.

### 2.4. Water Activity

Determination of water activity (aw) of tea samples was performed using the Water Activity Meter Fast-Lab in 3 replicates for each sample.

### 2.5. Chemical Reagents

#### 2.5.1. Total Polyphenolic Content and Total Antioxidant Capacity

Determination of the total polyphenolic content was performed with the use of the following reagents: Folin–Ciocalteu phenol reagent (Centralchem, Bratislava, Slovakia), sodium carbonate p.a. (99%; Centralchem, Slovakia), gallic acid (3, 4, 5-Trihydroxybenzoic acid monohydrate, 99%; Alfa Aesar, Thermo Fisher (Kandel) GmbH, Dreieich Germany). All the reagents were dissolved in distilled water. The necessary reagent used for the total antioxidant capacity measurements was 1,1-Diphenyl-2-picrylhydrazyl radical (DPPH) (Sigma-Aldrich; Merck KGaA, Darmstadt, Germany) dissolved in methanol p.a. (99.8%; Centralchem, Slovakia).

#### 2.5.2. HPLC Analysis

Caffeine standard (HPLC standard with a purity of 98% from Sigma-Aldrich GmbH, Steinheim, Germany), HPLC methanol (Chromasolv Gradient, purity ≥99.8%, Sigma-Aldrich GmbH, Steinheim, Germany), formic acid (ACS, purity ≥96%, Sigma-Aldrich GmbH, Steinheim, Germany), and deionized water ddH2O (18.2 MΩ cm^−1^, 25 °C) were used as standards and for mobile phase preparation.

### 2.6. Determination of Total Polyphenolic Content and Antioxidant Activity

#### 2.6.1. Apparatus

A double-beam spectrophotometer (T80 UV/VIS Spectrometer; PG Instruments Ltd., OK Service, Praha, Czech Republic) equipped with a cuvette holder for 8 cuvettes was used for determination of total polyphenolic content and total antioxidant activity. Glass cuvettes (type S/G/10; Exacta+Optech GmbH, Berlin, Germany) were used for analysis. The injected volume was 50 μL for TPC and 100 μL for TAC.

#### 2.6.2. Determination of Total Polyphenolic Content

Determination of total polyphenolic content (TPC) was realized by the modified Folin–Ciocalteu spectrophotometric method [13]. The principle of the method is in the reaction of polyphenols with Folin–Ciocalteu reagent, which provides a blue complex in alkaline conditions. The intensity of the blue color depends on the quantity of polyphenol compounds in the extract. In our case, the gallic acid was used as a standard where 0.1 g of gallic acid was weighed and diluted with demineralized water of up to 100 mL in volume to prepare a stock solution. From the prepared stock solution, 1 mL was taken and diluted with distilled water of up to 200 mL in volume. This solution was used to prepare the calibration curve in the calibration range of 5–200 mg.L^−^^1^ of gallic acid. Blank contained Folin–Ciocalteu reagent and distilled water without the standard or extract. The calibration curve had a correlation coefficient R^2^ = 0.999. The quantity of polyphenols was expressed in mg.100 g^−1^ as gallic acid equivalent (GAE). Quantities of 50 μL of tea extracts were put into 50 mL volumetric flasks, then 2.5 mL of Folin–Ciocalteu reagent diluted with distilled water (1:2 *v*/*v*) and 5 mL of Na_2_CO_3_ (20% water solution) were added, and flasks were filled up to 50 mL volume with distilled water. The flasks were let for 2 h at room temperature to develop a blue complex. All samples were measured in triplicate for each analysis, with 765 nm wavelengths.

#### 2.6.3. Determination of Total Antioxidant Capacity

Total antioxidant capacity (TAC) was determined as a free radical scavenging assay with the use of DPPH (2,2-diphenyl-1-picrylhydrazyl) according to Brand–Williams [14] with modification [15]. The amount of 0.025 mg of DPPH was weighed properly, then dissolved with methanol, and a volumetric flask with the stock solution was filled up to 100 mL. The stock solution was diluted with methanol at a ratio of 1:9 to obtain an absorbance of approx. 0.7. The diluted DPPH solution was put into glass cuvettes in the amount of 3.9 mL, and initial DPPH absorbance (A_0_) was measured at a wavelength of 515.6 nm. Then, 100 μL of sample extract was pipetted into a cuvette, and the mixture was stirred with a glass stick. Cuvettes were left in the dark for 10 min, and then final absorbance (A_t_) was read. The decrease in absorbance of the resulting solution was then measured spectrophotometrically at 515.6 nm (T80 UV/VIS Spectrometer). All the experiments were performed in triplicate. The scavenging capacity was calculated using the following Equation (1) and expressed as inhibition of DPPH:

(1) $$\%\;\mathrm{inhibition}\;\mathrm{of}\;\mathrm{DPPH}=\frac{{(A}_{0}-A_{s})-{\;(A}_{t}-A_{s})}{{(A}_{0}-A_{s})}\cdot 100$$

A_0_ is the initial absorbance of the DPPH solution, A_s_ is the absorbance of methanol 10 min after adding the tea extract.

### 2.7. Determination of Caffeine Using HPLC-DAD

Measurements were conducted using the HPLC Agilent Infinity 1260, manufactured by Agilent Technologies GmbH (Agilent Technologies GmbH, Waldbronn, Germany), equipped with a DAD detector (1260 DAD VL+). The separation was realized on a LiChroCART 250-4 Purospher STAR, with an RP-18 endcapped column (250 mm × 4 mm × 5 µm; Merck KGaA, Darmstadt, Germany). Methanol (A) and 0.1% solution of formic acid in ddH20 (*v*/*v*) (B) were used as a mobile phase. The separation was realized at gradient elution (0–2 min: 20% A + 80% B); in the 2–15 min period, the ratio of mobile phases was gradually changed to a final value of 40% A + 60% B, and the post time and equilibration were changed back to 20% A + 80% B. Equilibration time was 3 min, the flow rate was 1 mL min^−1^, and the injection volume was 3 µL. The column oven was set at 40 °C. The detection wavelengths were set at 240 and 280 nm. The spectral data obtained in this way were processed using the Agilent OpenLab ChemStation program.

### 2.8. Heavy Metals Determination—AAS

Exactly 1 g of homogenized tea sample was weighted on analytical balances. The weighed sample was placed in a mineralization cartridge and poured into 5 cm^3^ of redistilled water and 5 cm^3^ of concentrated nitric acid. The closed cartridge was mineralized in a MARS X-press microwave digestion machine (USA).

After the mineralization, the obtained minerals were filtered through MUNKTELL grade 390.84 g/m^2^ (green) quantitative filter paper into 50 mL volumetric flasks. They were mixed with distilled water up to the mark. The following was then measured using a VARIAN AA 240FS (Australia) under the mineralization conditions shown in Table 2. Used standard: multi-element standard for GF AAS (16 elements) Merck (Germany). Repeatability of determination in analysis deviation max 3%, gas flows: air: 13.5 L min^−1^, Acetylene: 2.0 L min^−1^. Mercury was measured in the sample directly without treatment on an AMA 254 instrument (Czech Republic) by atomic absorption spectrometry using the mercury vapor generation technique. Wavelength Hg—253.65 nm; detection limit—1.5 ng kg^−1^ dry matter. Conditions of AAS are shown in following Table 3.

### 2.9. Statistical Analysis

For the summarizing and describing of our results, descriptive statistics were used. Parameters such as minimum, maximum, arithmetic means were used for interpreting the results. To discover any possible significant differences between the analyzed samples, the ANOVA Duncan test and REGWQ were used. This statistical analysis was performed using Microsoft Office Excel 365 for iOS. To create a correlation for bioactive compounds and heavy metals, Pearson’s correlation test was used. To create a model that would be useful for determining the geographical origin, Linear Discriminant Analysis was used (XL Stat, Addinsoft for iOS).

## 3. Results and Discussion

### 3.1. Changes in Total Antioxidant Capacity, and the Content of Polyphenols and Caffeine, in Teas with Regard to Their Fermentation Process and Origin

**Figure 1:**
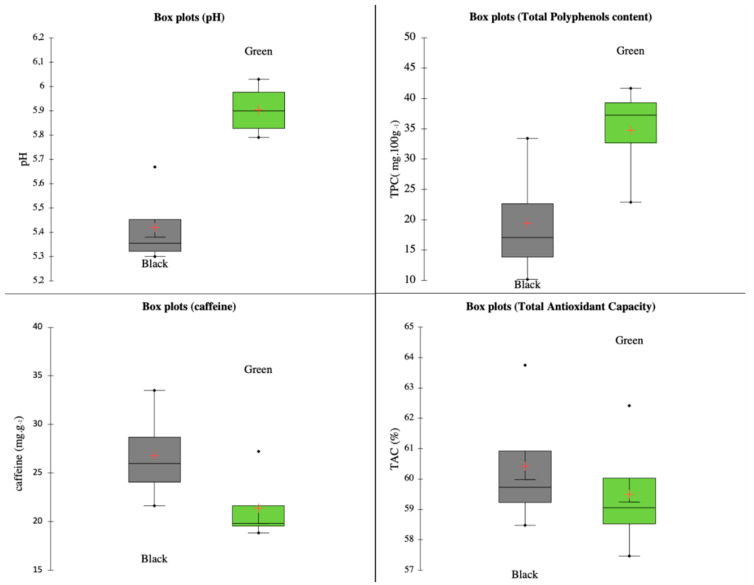
Boxplots of TAC, TPC, caffeine content, and pH in relation to the fermentation process.

Using the ANOVA Duncan and REGWQ test, the green and black types of each sample were compared. This comparison of the fermentation processes is shown in Table 4, and supporting boxplot visualizations are shown in Figure 1.

As depicted in Table 4, water activity shows significant differences in each pair of samples. In general, the water activity of green teas showed values from 0.531 to 0.580, while black teas showed slightly lower values, from 0.543 to 0.551. The same can be concluded regarding the percentage of moisture.

Analyzed green samples showed pH values from 5.79 to 6.03. On the contrary, samples of black teas ranged from 5.3 to 5.67. Accordingly, Zhu et al. [16] proved that the pH of green tea infusion is from 5.83 to 6.39 and that of black tea is from 4.96 to 6.15. Zhank et al. (2017) [17] proved that this parameter might be affected by water type. Furthermore, both the Duncan and REGWQ tests proved that the fermentation process caused a significant difference in this parameter (Table 4).

Green tea (*Camellia sinensis* L.) is considered a dietary source of antioxidant compounds, especially polyphenolic components. Highly abundant are epicatechin, epicatechin-3-gallate, epigallocatechin, and epigallocatechin-3-gallate. These polyphenols are effective scavengers of oxygen radicals [18]. Polyphenols’ content depends on the genetic background and growing conditions (such as temperature, nitrogen availability, and light conditions) of the plant material [19]. Moreover, our findings proved that the total content of polyphenols depends on the fermentation process, even when infusions are prepared from leaves obtained from the same plant. This fact may be explained by assuming that the heat-labile enzyme polyphenol oxidase activity in tea is reduced during fermentation by heating with steam. Therefore, as Yan et al. [20] reported, green tea may contain more polyphenolic compounds. However, the tea leaf processing technology might influence its final properties [21]. This supports our findings, given that we determined that green teas contained more polyphenolic compounds, ranging from 22.951 to 41.789 g GAE kg^−1^, whereas the contents in black teas were from 10.203 to 33.381 g GAE kg^−1^. Thus, the Duncan and REGWQ tests showed significant differences between green and black tea within each pair of samples with regard to the content of polyphenols.

On the other hand, the total antioxidant capacity of each pair showed significant differences only within samples 2 (Japan) and 4 (Korea), but the other two pairs reached almost equal values (Table 4). The analyzed green samples reached TAC values from 57.565% to 62.228%, and the black tea samples reached slightly higher values, from 58.599% to 63.739%. As reported, black tea has a higher antioxidant content [22], which is in accordance with our findings. Furthermore, we detected that both types of teas (black and green) showed a negative correlation (Pearson’s correlation coefficient—0.797) between TPC and TAC values; this was previously reported by Chang et al. [23]. This fact may be explained given that at a higher temperature of the fermentation process, degradation or change in chemical structure can occur and eventually change the structure of temperature-sensitive molecules, which also have antioxidant potential. Generally, most natural antioxidants are multifunctional, meaning that the total antioxidant capacities of the food matrix cannot be fully described by one single method [24].

Salihović et al. [25] determined caffeine content in commercially available green and black tea, and their results showed that the caffeine content in the green ones was in the range of 33.90–110.73 (mg g^−1^). Our samples of green tea showed values of caffeine ranging from 18.831 to 27.224 mg g^–1^. Even though caffeine is stable during the fermentation process, some suggest that black tea contains more caffeine than green tea obtained from the same plant. Researchers suggest that several factors influence caffeine content in tea, and the primary factor is the level of oxidization process that leaves undergo. Black tea is 100% processed, while green tea is typically processed only up to 40%. Our obtained values support the hypothesis that black tea showed, on average, a higher content of caffeine, which is accordance with the findings of Heckman et al. [26]. The increase in caffeine content in black tea samples varies from 1% (sample 1 with values almost identical) to 53% (sample 3—Nepal tea), suggesting that agricultural differences may also play a role. However, it is essential to add that based on the ANOVA (Table 4), all samples show significant differences in caffeine content according to the type of tea fermentation.

To determine whether the parameters mentioned above show a potential to be used for purposes of geographical origin identification, values were subjected to Principal Component Analysis and Linear Discriminant Analysis. Both proved that a model suitable for geographical origin identification could not be computed (data not shown) based only on the parameters mentioned above (Rao’s approximation *p*-value > 0.05), meaning that these parameters did not represent sufficient variability regarding the geographical origin.

### 3.2. Changes in Heavy Metal Content in Relation to Fermentation Process and Origin

In response to increased soil pollution, many have raised concerns about the health issues of food products containing high concentrations of certain heavy metals, including copper (Cu), zinc (Zn), lead (Pb), cadmium (Cd), chromium (Cr), and nickel (Ni). Due to this, regulatory limits for metals in tea leaves or herbal materials had been developed in different countries (Li et al., 2020). Even though heavy metals in food are very complex phenomena, we focused our research on the identification of cadmium (Cd), lead (Pb), copper (Cu), zinc (Zn), cobalt (Co), chromium (Cr), nickel (Ni), manganese (Mn), and iron (Fe). Moreover, various other scientific papers proved that the excessive levels of heavy metals in tea and their subsequent absorption in the human body might cause poisoning and various health problems. The concentration of heavy metals obtained from green and black tea are shown in Table 5.

Tea contains minerals such as potassium, manganese, boron, selenium, strontium, zinc, and copper. Some of these may have a crucial role in the activation of certain enzymes. Tea plants uptake heavy metals from the soil, which may cause their accumulation in edible parts [27,28].

The mean trace element concentration copied the following decreasing order: Mn > Fe > Cu > Zn > Ni > Cr > Pb > Co > Cd > Hg. Copper is essential for the human body; however, high concentrations may cause health problems, such as kidney failure. The concentration of Cu in our samples of green tea was in intervals of 5.3 to 13.8 mg kg^−1^, and in black teas, the concentration was from 7.5 to 16.8 mg kg^−1^. In both cases, the highest values were observed in samples from China (Chinese legislative limitation for Cu is 60 mg kg^−1^) [29]. Zinc showed a homogenous concentration range from 24.0 to 31.8 mg kg^−1^.

According to [30], manganese is the only mineral found in sufficiently substantial amounts in teas to cover daily requirements. In this study, black tea reached the highest concentrations for this mineral. High levels of manganese may interfere with iron absorption and result in ADHD-like symptoms in children exposed in utero [31]. Our samples ranged from 247.7 to 553.9 mg kg^−1^. The World Health Organization reported arsenic (As), lead (Pb), mercury (Hg), and cadmium (Cd) as chemicals that cause major health concerns. Chromium, cobalt, and lead reached relatively similar values; on average, 1.1, 0.61, and 0.94 mg kg^−1^, respectively. On the other hand, the lowest concentration was measured in mercury (Hg). Relatively similar concentrations were observed in a previous study [32].

**Figure 2:**
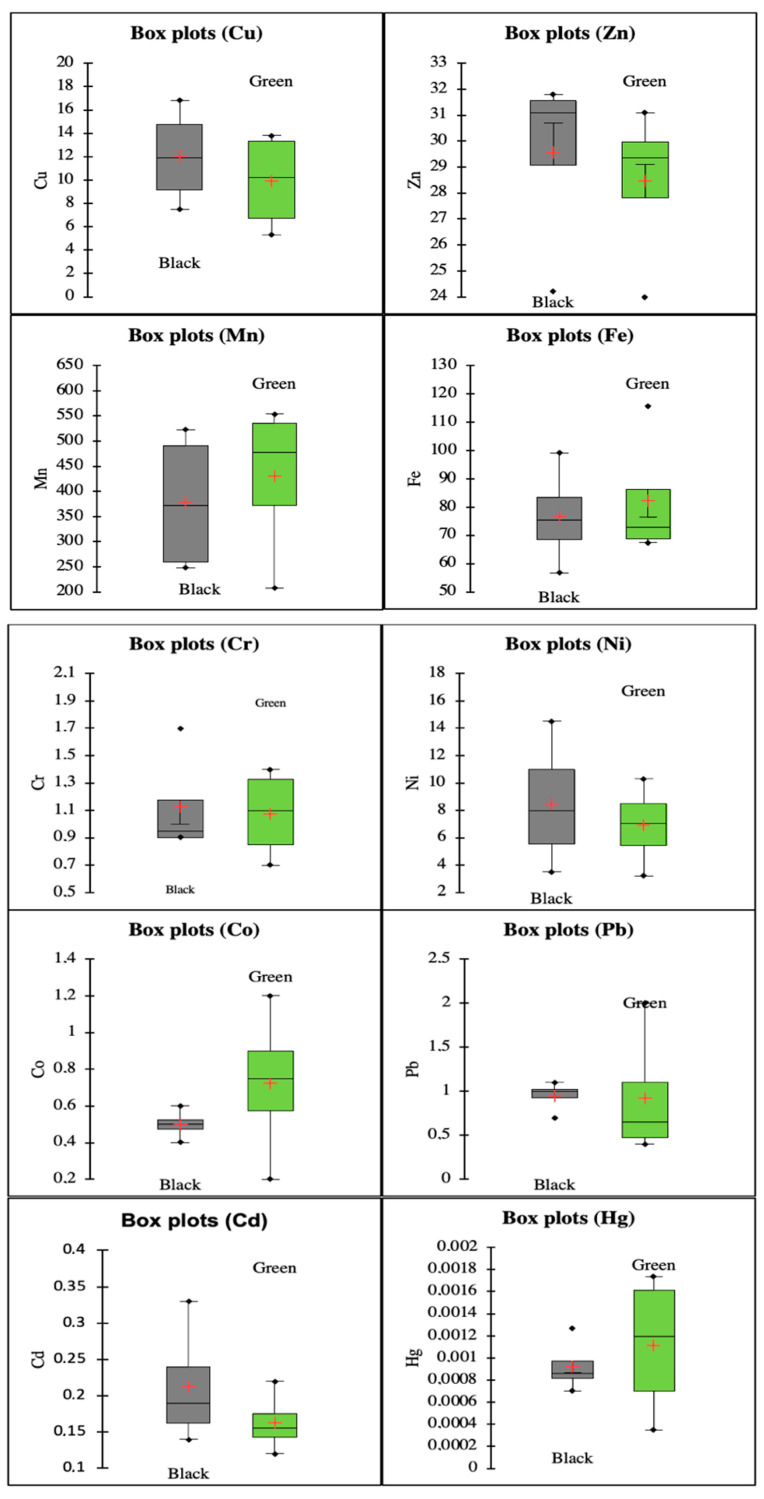
Boxplots of heavy metal concentration in relation to the fermentation process used.

To observe any significant differences regarding the heavy metal content and fermentation process, the ANOVA Duncan test and REGWQ were performed. Results are shown in Table 6. These tests proved that there is no statistical difference (*p* > 0.05). Supporting boxplot visualizations are shown in Figure 2.

**Figure 3:**
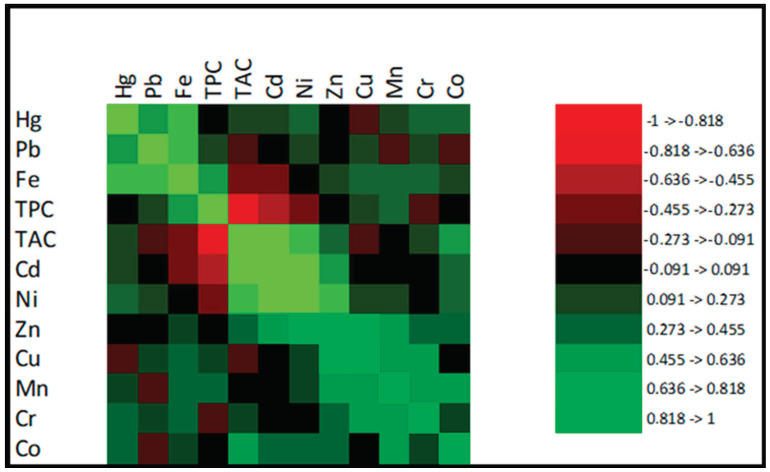
Correlation map for heavy metals, TAC, and TPC in tea samples.

Various publications raised concerns about heavy metals and their possible effect on bioactive substances. Based on Table 5, we also detected those that may interfere with the bioactivity of aqueous soluble compounds in tea. To observe any possible correlation with the content of individual heavy metals, Total Antioxidant Capacity, and Total Polyphenol Content, data were subjected to Pearson’s correlation (Figure 3).

Figure 3 showed that Cd had a negative correlation (−0.636 to −0.455) with Total Polyphenol Content. The research focused on antioxidant capacity, metal contents, and their health risk assessment of Tartary Buckwheat Teas similarly observed a negative correlation of Cd and total polyphenols content [27]. On the other hand, we observed a strong positive correlation with the content of Cd stress that affected the increase in Total Antioxidant Capacity. Similar findings were reported by [33], who claimed that cadmium increased the content of flavonoids and antioxidant capacity. Our findings may suggest that certain antioxidants play a role in the cadmium defense. Therefore, antioxidant capacity, measured as free-radical scavenging ability, was increased. Similar correlations were observed with TAC, TPC, and Ni content. However, based on Figure 3, these were not as strong as in the previous case but still observable. Lead showed a weak positive correlation (0.273–0.455) with TPC. A similar fact was observed by Li et al., 2020 [27]. Mercury, zinc, and cobalt did not correlate with TPC. However, only weak correlations were observed with TAC. In addition, copper did not show a strong correlation neither with TPC, nor with TAC.

**Figure 4:**
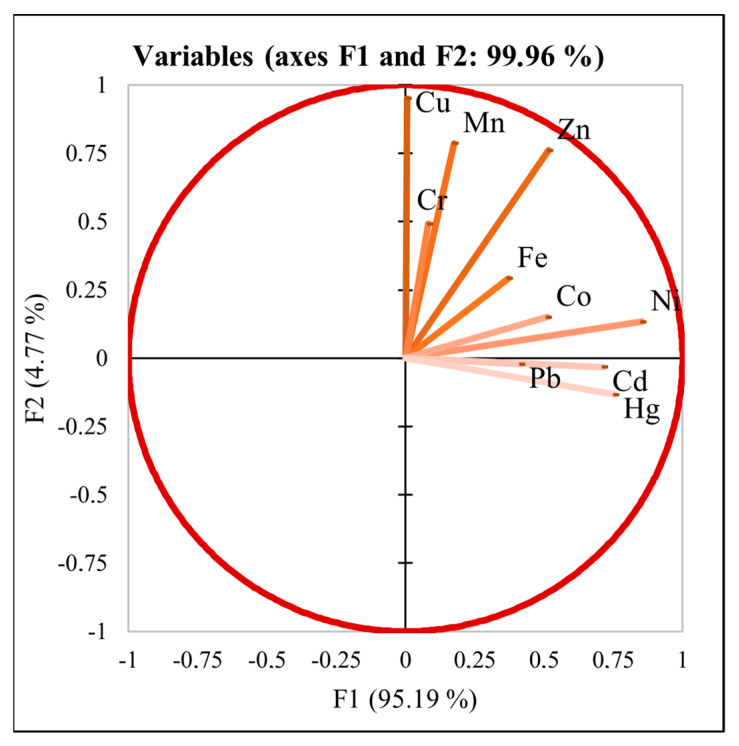
Representation of variables related to geographical origin based on heavy metal content.

**Figure 5:**
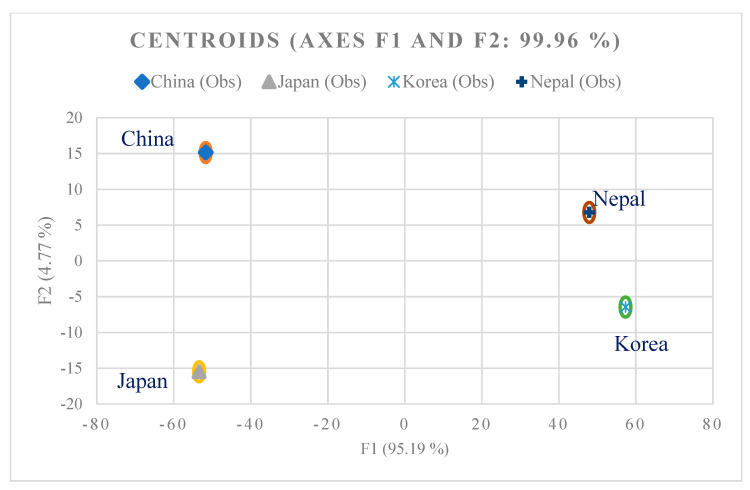
LDA map of geographical origin based on heavy metal content.

Chemical fertilizers containing heavy metals and pesticide use along with industrial activities have been found to cause enrichment of soil in tea gardens with heavy metals such as Cd, Pb, As, Cr, Hg, Mn, and Cu. The authors of [34] suggested the detection of changes in heavy metal contents in tea in relation to the country of origin and the study of any possible observable patterns. To do so, we subjected our data to Linear Discriminant Analysis (LDA), which created a linear combination of characteristics in order to separate two or more classes of observations. With *p* < 0.0001, the Wilks’ Lambda test (Rao’s approximation) shows that the vectors are not the same. Figure 4 shows that the initial variables correlate with two factors, F1 and F2, meaning that F1 and F2 explain 99.96% of the variability between these regions. From Figure 4, it is evident that the F1 factor explains the majority (95.19%) of variability between teas from Japan, China, Korea, and Nepal. Furthermore, we observed that heavy metals, such as Hg, Cd, Pb, Ni, Co, and Fe, correlate with F1. Moreover, Figure 5 confirms that countries of origin (China, Japan, Nepal, and Korea) are well separated, and there is no visible overlay (Figure 5). Our results confirmed the theory of [11] that heavy metals may possibly be used as a discriminant for the identification of the geographical origin of tea.

The confusion matrix, calculated for the training samples based on heavy metals for all four observed geographical groups, equals 100%, meaning that all training samples were identified correctly using LDA (data not shown). Furthermore, LDA calculates membership probabilities for unknown samples using the Cross-validation: Prior and posterior classification, and membership probabilities (Table 7). Our results proved that LDA using heavy metal content correctly authenticated the origin of teas from Japan, China, and Nepal but did not identify the origin of Korean samples. These samples were misidentified as Nepal and Japan tea samples, respectively. Given that the prior and posteriors classification showed two misclassified samples (Table 7), the confusion matrix for the cross-validation was lowered by 25% to the total accuracy of the LDA model of 75% (Table 8).

## 4. Conclusions

By comparing fermented and non-fermented teas of a single plant origin, our study proved that the fermentation process could significantly affect the content of bioactive compounds, especially the content of polyphenolic compounds, and therefore, possibly affect the antioxidant potential of tea. However, geographical origin might also play a role in terms of the content of bioactive compounds.

The content of heavy metals, as a parameter which is nowadays closely monitored, showed that the mean trace element concentration followed this order: Mn > Fe > Cu > Zn > Ni > Cr > Pb > Co >Cd > Hg. Furthermore, we proved that their content is not affected by the fermentation process. However, geographical origin affects their content significantly. Based on this premise, we created an LDA model that clearly distinguished between regions of origin. As shown, 95.19% of the variability was explained with Co, Ni, Cd, Pb, and Hg. This means that these heavy metals are the most significant ones with regard to the identification of geographical origin. LDA proved 75% accuracy when only Korean samples were misclassified. These findings suggest that heavy metal contents could be reliable markers for health-related issues and authentications of tea origin.

## Figures and Tables

**Table 1:** Characterization of tea samples.

Fermentation	Sample ID	Origin	Type
Non-fermented	1A	China	Green
Fermented	1B	China	Black
Non-fermented	2A	Japan	Green
Fermented	2B	Japan	Black
Non-fermented	3A	Nepal	Green
Fermented	3B	Nepal	Black
Non-fermented	4A	Korea	Green
Fermented	4B	Korea	Black

**Table 2:** Parameters of the mineralization process.

Phase	Power (W)	Power (%)	Building Up Time (min)	Temperature (°C)	Hold-Off (min)
Initialization (Achieving the specified conditions)	800	90	15	160	0
Mineralization (Maintaining the specified conditions)	800	90	0	160	20
Cooling	–	–	–	–	20

**Table 3:** Condition of Atomic Absorption Spectroscopy measurements of heavy metals.

Heavy Metal	Detection Limit (mg L^−1^)	Sensitivity (mg L^−1^)	Wave-Length (nm)
Cd	0.001	0.01	228.8
Pb	0.02	0.1	217.0
Cu	0.002	0.03	324.8
Zn	0.006	0.008	213.9
Co	0.005	0.05	240.7
Cr	0.003	0.04	357.9
Ni	0.008	0.06	232.0
Mn	0.003	0.02	279.5
Fe	0.005	0.04	241.8

**Table 4:** ANOVA Duncan and REGWQ comparison of selected parameters of individual samples in relation to the fermentation process.

**Sample 1**						
	**aw**	**Moisture (%)**	**pH**	**TPC m (g GAE kg^−1^)**	**TAC (%)**	**Caffeine (mg g^−1^)**
China (green)	0.580 ^a^	7.247 ^b^	6.030 ^a^	41.798 ^a^	59.178 ^a^	27.224 ^a^
China (black)	0.551 ^b^	8.440 ^a^	5.330 ^b^	19.083 ^b^	59.474 ^a^	27.095 ^b^
Pr > F(Model)	<0.0001	<0.0001	<0.0001	<0.0001	0.469	0.007
Significant	Yes	Yes	Yes	Yes	No	Yes
**Sample 2**						
	**aw**	**Moisture (%)**	**pH**	**TPC (g GAE kg^−1^)**	**TAC (%)**	**Caffeine (mg g^−1^)**
Japan (green)	0.543 ^b^	7.720 ^b^	5.953 ^a^	36.052 ^a^	57.565 ^b^	18.831 ^b^
Japan (black)	0.551 ^a^	9.682 ^a^	5.303 ^b^	15.173 ^b^	59.856 ^a^	21.650 ^a^
Pr > F(Model)	<0.0001	<0.0001	<0.0001	<0.0001	<0.0001	<0.0001
Significant	Yes	Yes	Yes	Yes	Yes	Yes
**Sample 3**						
	**aw**	**Moisture (%)**	**pH**	**TPC (g GAE kg^−1^)**	**TAC (%)**	**Caffeine (mg g^−1^)**
Nepal (green)	0.531 ^b^	7.820 ^b^	5.788 ^a^	38.706 ^a^	58.778 ^a^	19.788 ^b^
Nepal (black)	0.551 ^a^	8.028 ^a^	5.670 ^b^	33.381 ^b^	58.599 ^a^	36.715 ^a^
Pr > F(Model)	<0.0001	<0.0001	<0.0001	<0.0001	0.683	<0.0001
Significant	Yes	Yes	Yes	Yes	No	Yes
**Sample 4**						
	**aw**	**Moisture (%)**	**pH**	**TPC (g GAE kg^−1^)**	**TAC (%)**	**Caffeine (mg g^−1^)**
Korea (green)	0.547 ^b^	8.488 ^b^	5.842 ^a^	22.951 ^a^	62.228 ^b^	19.775 ^b^
Korea (black)	0.562 ^a^	9.628 ^a^	5.383 ^b^	10.203 ^b^	63.739 ^a^	24.852 ^a^
Pr > F(Model)	<0.0001	<0.0001	<0.0001	<0.0001	0.002	<0.0001
Significant	Yes	Yes	Yes	Yes	Yes	Yes

Notes: ^a^, ^b^ index = the values designated by the different letters in the columns are significantly different (*p* < 0.05).

**Table 5:** The concentration of heavy metals in green and black teas.

**Sample ID**	**Type**	**Country of Origin**	**Cu**	**Zn**	**Mn**	**Fe**	**Cr**
1A	Green	China	13.8	31.1	528.5	69.3	0.9
2A	Green	Japan	5.3	24.0	207.6	67.5	0.7
3A	Green	Nepal	13.2	29.6	427.6	115.6	1.4
4A	Green	Korea	7.2	29.1	553.9	76.5	1.3
1B	Black	China	16.8	30.7	522.5	72.6	1.7
2B	Black	Japan	7.5	24.2	263.8	78.2	1.0
3B	Black	Nepal	14.1	31.5	480.1	99.1	0.9
4B	Black	Korea	9.7	31.8	247.7	56.7	0.9
**Sample ID**	**Type**	**Country of Origin**	**Ni**	**Co**	**Pb**	**Cd**	**Hg**
1A	Green	China	6.2	0.8	0.4	0.15	0.000345
2A	Green	Japan	3.2	0.2	0.8	0.12	0.000817
3A	Green	Nepal	7.9	0.7	2.0	0.16	0.001737
4A	Green	Korea	10.3	1.2	0.5	0.22	0.001572
1B	Black	China	6.2	0.4	0.7	0.17	0.000702
2B	Black	Japan	3.5	0.5	1.0	0.14	0.000868
3B	Black	Nepal	9.8	0.5	1.1	0.21	0.001271
4B	Black	Korea	14.5	0.6	1.0	0.33	0.000854

Note: Concentration values are present as mg kg^−1^.

**Table 6:** ANOVA Duncan and REGWQ comparison of average concentration of heavy metals in tea in terms of their fermentation processes.

	**Cu**	**Zn**	**Mn**	**Fe**	**Cr**
**Black**	12.025 ^a^	29.550 ^a^	378.525 ^a^	76.650 ^a^	1.125 ^a^
**Green**	9.875 ^a^	28.450 ^a^	429.400 ^a^	82.225 ^a^	1.075 ^a^
Pr > F(Model)	>0.05	>0.05	>0.05	>0.05	>0.05
Significant	No	No	No	No	No
Pr > F(Type)	>0.05	>0.05	>0.05	>0.05	>0.05
Significant	No	No	No	No	No
	**Ni**	**Co**	**Pb**	**Cd**	**Hg**
**Black**	8.500 ^a^	0.500 ^a^	0.950 ^a^	0.213 ^a^	0.001 ^a^
**Green**	6.900 ^a^	0.725 ^a^	0.925 ^a^	0.163 ^a^	0.001 ^a^
Pr > F(Model)	>0.05	>0.05	>0.05	>0.05	>0.05
Significant	No	No	No	No	No
Pr > F(Type)	>0.05	>0.05	>0.05	>0.05	>0.05
Significant	No	No	No	No	No

Notes: ^a^ index = the values designated by the different letters in the columns are significantly different (*p* < 0.05).

**Table 7:** Cross-validation: Prior and posterior classification, membership probabilities, scores, and squared distances.

Observation	Prior	Posterior	China	Japan	Korea	Nepal
1A	China	China	1.000	0.000	0.000	0.000
2A	Japan	Japan	0.000	1.000	0.000	0.000
3A	Nepal	Nepal	0.000	0.000	0.000	1.000
4A	Korea	Nepal	0.000	0.000	0.000	1.000
1B	China	China	1.000	0.000	0.000	0.000
2B	Japan	Japan	0.000	1.000	0.000	0.000
3B	Nepal	Nepal	0.000	0.000	0.000	1.000
4B	Korea	Japan	0.000	1.000	0.000	0.000

**Table 8:** Confusion matrix for the cross-validation results.

From\To	China	Japan	Korea	Nepal	Total	% Correct
China	2	0	0	0	2	100.00%
Japan	0	2	0	0	2	100.00%
Korea	0	1	0	1	2	0.00%
Nepal	0	0	0	2	2	100.00%
Total	2	3	0	3	8	75.00%
